# Community knowledge, attitude, and practices towards tuberculosis in Shinile town, Somali regional state, eastern Ethiopia: a cross-sectional study

**DOI:** 10.1186/1471-2458-14-804

**Published:** 2014-08-07

**Authors:** Daniel Tolossa, Girmay Medhin, Mengistu Legesse

**Affiliations:** Department of Medical Laboratory Technology, Jig-jiga Health Sciences College, P.O. Box 504, Jig-jiga, Ethiopia; Aklilu Lemma Institute of Pathobiology, Addis Ababa University, P.O. Box 1176, Addis Ababa, Ethiopia

**Keywords:** Tuberculosis, KAP, Shinile town

## Abstract

**Background:**

Though tuberculosis (TB) is preventable and curable, its global burden remains enormous. Similarly, TB is one of the major public health problems in Ethiopia, particularly in geographically isolated areas like Shinile town. The people in Shinile town, Somali Regional State of Ethiopia, are underserved in all forms of health care and suffer from high burden of TB. Low level of knowledge about TB could affect the health-seeking behavior of patients and sustain the transmission of the disease within the community. Therefore, the current study was undertaken in Shinile town with the objective of assessing communities’ knowledge, attitude and practices towards TB.

**Methods:**

Community-based cross-sectional survey, involving 410 randomly selected individuals, was conducted in Shinile town from January to May, 2013. Data were analyzed using STATA V.11. Logistic regression technique was used to determine the association between socio-demographic characteristics and communities’ knowledge of TB.

**Results:**

While 94.9% of the respondents said that they ever heard about TB, only 22.9% knew that TB is caused by bacteria. Eighty percent have awareness that TB can be transmitted from a patient to another person and 79.3% know that transmission of TB can be preventable. Persistence cough (72.4%) was the most commonly stated symptom of TB and modern drugs used in health institutions (68.1%) was the preferred choice of treatment. Two hundred and ninety one respondents (71.0%) said that they would seek treatment at health facility if they realized that they had symptoms related to TB. Two hundred and twenty seven respondents (55.4%) considered TB as a very serious disease and 284 (69.3%) would experience fear if they themselves had TB. Individuals with educational level of grade 8 up to grade 12 had increased odds of having good level of overall TB knowledge compared to illiterate individuals (OR = 2.3; 95% CI: 1.2 to 4.6).

**Conclusion:**

The communities in Shinile town have basic awareness about TB which is not translated into the knowledge about the cause of the disease. Therefore, health education directed towards bringing a significant change in the knowledge of TB must be stepped-up within the TB control program.

**Electronic supplementary material:**

The online version of this article (doi:10.1186/1471-2458-14-804) contains supplementary material, which is available to authorized users.

## Background

Tuberculosis remains a major global health problem [1, 2]. It causes ill-health among millions of people each year and ranks as the second leading cause of death from an infectious disease worldwide, after the human immunodeficiency virus (HIV) [1]. World Health Organization (WHO) estimated the global burden of disease caused by TB in 2011 as follows: 8.7 million incident cases, 12 million prevalent cases and 1.4 million deaths [1]. Most of the estimated number of cases in 2011 occurred in Asia (60%) and Africa (24%) [1]. The incidence of TB has more than doubled in Africa during the last two decades [3]. TB is also one of the major diseases that cause enormous economic crisis in low income countries [4].

TB is one of the major causes of morbidity and mortality in the Horn of Africa with Ethiopia carrying a heavy burden [5]. In Ethiopia, TB is the major cause of death and hospital admission [6]. The country is one of the top three in Africa and ranked 9th among the 22 countries in the world with a high-burden of TB in 2010 [7]. The disease is also one of the major health problems in Somali Regional State (SRS) of Ethiopia. The people in the region are not only exceedingly poor, but also bear a disproportionately high burden of TB [8]. According to the Somali Regional State Health Bureau (SRSHB) report, the disease is in the top ten causes of outpatient visit, hospital admission and deaths in the region in 2008 [SRSHB, 2008-Unpublished]. Similarly, being geographically isolated and remote population with poor infrastructure and communication, the people in Shinile town is underserved in all forms of health care and is perceived as low status.

Factors such as infection with HIV, poor nutritional status, smoking, increased susceptibility of infants and the elderly and increased virulence and/or increased dose of bacilli have been identified as substantial contributors for the development of the disease and its epidemiological burden [4, 9, 10]. Poverty and lack of awareness about TB are also considered the most important factors that increase the risk of exposure to TB [11]. In addition, poor access to health facilities [12–15], lack of financial source [16–18] and lack of knowledge about the cause, mode of transmission, and symptoms, as well as appropriate treatment of TB within communities [14–16] do not only affect the health seeking behavior of patients that favors the use of traditional healers over biomedical approaches, but also could contribute to poor adherence to TB treatment and/or long delay in diagnosis, which pose a formidable challenge to control the disease [14, 15, 18]. The use of traditional medicine not only delays health-seeking behavior, but it also presents ample time for the infection to spread to the healthy population [19–21]. Furthermore, when the community holds a strongly negative concept of TB, this can negatively influence the social relations and the moral identity of those afflicted by the disease and also efforts to control TB in general [16, 22, 23]. For these reasons, creating general awareness about the TB among communities and initiating community participation in the control of the disease make up one component of the six basic components of the “Stop TB Strategy” of the WHO [24].

Little is known about the knowledge, attitude and practices of communities towards TB in the current study area. Therefore, the study was conducted in Shinile town, SRS, eastern Ethiopia to assess community’s knowledge, attitude and practice towards TB.

## Methods

### Study area

The current study was conducted in Shinile town, SRS, eastern Ethiopia between January and May, 2013. The SRS is one of the nine regional states that constitute the federal democratic republic of Ethiopia [25]. Geographically, the region occupies a large area and falls in the eastern and southeastern part of the country with land mass area of about 350,000 km^2^ and an estimated total population of 4,445,219 people [25]. 86.1% of the population resides in rural areas while only 13.9% reside in urban areas [25]. Hence, this implies the backbone of the region's economy depends on livestock, as most of the rural dwellers are pastoralists and agro-pastoralists [25]. The people in the region are among the poorest in Ethiopia, and thus they are disproportionately affected by TB. The annual case rate of smear positive pulmonary TB in the year 2011 was reported to be 30% [26]. Similarly, the annual case detection rate of Pulmonary TB was noted to be 10.3% which is much lower than the national rate of 36.8% [26]. Shinile town is located in the south western part of the region, at a distance of about 155 km west of Jig-jiga, the capital city of SRS and 555 km east of Addis Ababa. The town has one Kebele, in which there are eight villages (Menders). According to the Shinile Woreda’s Health Bureau, the current total population of the Shinile Woreda is about 54,947, of which 10,609 (male = 5,899 and female = 4,710) are residents of Shinile town.

### Study design, source population and study participants

The study design was a community-based cross-sectional and the data was collected using questionnaire administered by data collectors. Every individual aged 18 years and above residing in the randomly selected four villages of the study town was eligible for the study. Study participants were all 18 years of age and older individuals who were randomly selected from eligible individuals in the selected households and consented to participate in the study. Individuals were not included if they were guests, if they were less than 18 years of age, if they did not consent and if they were mentally ill.

### Sample size and sampling technique

The required sample size was calculated using the formula required for determination of sample size for estimating single proportion [27]. Based on the assumption that 50% of the study participants had high level of knowledge of TB and with additional assumption of 95% confidence interval, 5% margin of error and 10% non-respondent rate in our estimate, a total sample of 422 were needed. During sampling, 4 out of the 8 villages in the town were selected using simple random sampling technique and the calculated sample was proportionally distributed to the selected villages based on their number of households. Then, from each of the selected villages, households were selected using systematic random sampling technique. Finally, from all the eligible respondents in a household, only one was selected randomly for the interview. But in the absence of eligible respondent in a given household, a substitution was made by an individual in the next household.

### Data collection and quality control

The data collection tool was a standardized questionnaire (Additional file 1) which consists of questions on socio-demographic characteristics of the study participants and their knowledge, attitudes and practices towards TB. The questionnaire was first designed in English based on WHO guidelines [28] and information from different literatures developed for similar purpose [29–31]. Then the questionnaire was translated to Somali (the local language of the study area).

The questionnaire was pre-tested on randomly selected individuals from the survey area and these individuals were not participated in the main study. During the pre-test, the questionnaire was assessed for its clarity/understandability, reliability, sensitivity of the subject matter and for cultural acceptability in the area.

The data was collected through a face to face interview conducted by trained data collectors (students of clinical nursing, midwifery nursing and medical laboratory technology) recruited from apprenticeship attending students of Jig-jiga Health Science College. Each interview was made by a house-to-house visit and the participants were interviewed in their local language. One supervisor who speaks local language and had experience in data collection in previous similar studies was recruited to monitor the entire process.

### Variables considered in this study

The independent variables included in the data analysis were age, sex, marital status, ethnicity, religion, family income, and educational status. There were three main outcome variables, namely, knowledge of community towards TB, attitude of the community towards TB, and practice of the community towards TB [16, 29, 32, 33]. These three outcome variables are composite scores as it is not possible to measure them directly. Each of the three outcomes was measured using responses given by study participants to several set of questions. The detail of the content of the questions and how these outcomes were scored is summarized below:

### Knowledge of community towards tuberculosis

It was measured in two categories: overall knowledge and four subscales of knowledge of TB. The overall knowledge of the study participants about TB was assessed using the following 8 main questions: (1) able to mention bacteria/germ as a cause of TB, (2) able to mention the correct sign/symptoms of TB (persistent cough for two or more weeks, sputum with blood, chest pain, weight loss, loss of appetite and fever/sweat), (3) able to classify TB as a transmissible disease, (4) able to enumerate correct mode of transmission of TB (cough/breath, sharing cups, not sharing feeding materials, not through body contact or sharing clothes), (5) knowing that TB is treatable, (6) knowing that effective treatment for TB is modern drugs, (7) knowing that TB is preventable, and (8) able to enumerate correct preventive methods of TB (covering mouth and nose when coughing or sneezing, avoid sharing cups, using separate rooms, early treatment, not avoiding body contact/hand shaking). Each question was rated in such a way that a score of one was given to correct responses and a score of zero was used for incorrect/don’t know responses. Then, the responses to these questions were added together to generate a knowledge score ranging from 0 to 20. Finally, the composite score was dichotomized using mean (which was 10.67) as a cut-off value so that score above mean value was coded as 1 showing high overall knowledge of TB and score below mean value was coded as 0 showing low overall knowledge of TB in the community. Similarly, scores were generated for the four subscales of knowledge of TB (signs/symptoms, mode of transmission, effective treatment and preventive methods of the disease) and categorized into high and low knowledge of each domain using mean value.

### Attitude and practice of community towards tuberculosis

Attitude of the study participants about TB was assessed using the following questions: (1) able to consider TB as a very serious or somewhat serious disease and problem, (2) able to acknowledge that TB can happen to anybody, (3) able to mention positive feeling towards the disease and (4) feeling compassion and desiring to help the patient. An answer consistent with the correct attitude towards the disease and those infected was scored with one point. An answer not consistent with the correct attitude towards the disease and those infected was scored with zero point. Then, the responses to these questions were added together to generate an attitude score ranging from 0 to 7. Finally, the composite score was dichotomized using mean (which was 3.76) as a cut-off value so that score above mean value was coded as 1 showing good attitudes and score below mean value was coded as 0 showing poor attitudes.

Similarly, communities’ health care seeking behaviors and practices about TB was measured using the answers for the following 4 questions: (1) communities’ consultation to doctors or other health professionals about their illness if they had TB, (2) preference and reliance of the communities on the existing method of treatment, (3) seeking medical help immediately as soon as they realize they had symptoms related to TB or when symptoms that look like TB signs lasts for 2 or more weeks and (4) if the community supports and helps TB patient. An answer consistent with the correct health care seeking behaviors and practices with regards to the disease was scored with one point. An answer not consistent with the correct health care seeking behaviors and practices with regards to the disease was scored with zero point. Then, the responses to these questions were added together to generate a practice score ranging from 0 to 4. Finally, the composite score was dichotomized using mean (which was 2.4) as a cut-off value so that score above mean value was coded as 1 showing good practice and score below mean value was coded as 0 showing poor practices.

### Data processing and analysis

After the data was organized and edited to allow computer entry of all responses, EpiData V.3.1 was used for data entry and STATA V.11 was used for data analysis. Before data entry, manual checks of data cleaning was used to catch incorrect skip patterns, unreadable marks on the questionnaires, wrong codes and blank questions. Accordingly, inappropriate items, and inconsistent or unusable responses or items for which respondents have selected conflicting answers were modified in the field. Manually checked data were computerized using the EpiDataV.3.1. Counts or frequencies for each response were run and evaluated for the presence of missing responses and, a consistently documented decision was made so that the following items were regarded as missing: values that are implausible, impossible and/or are inconsistent with other information gathered in the survey interview and items that should have been completed during the course of data collection but were not, due to different reasons. The computerized data was transferred to STATA software version 11. Descriptive statistics using table of frequency distribution was used to summarize socio-demographic characteristics and the level of knowledge, attitudes and practices towards TB. Pearson chi-square test was used to evaluate the statistical significant of bivariate association of gender and selected covariate with the outcome variables. Bivariate and multivariable logistic regression was used to assess the effect of background characteristics of study participants on the level of overall knowledge of TB [16, 29, 32, 33]. Results were reported as statistically significant whenever p-values were less than 5%. Odds ratio (OR) was used to report strength of association between background variables and the target outcome variables.

### Ethical considerations

Approval to conduct the study was obtained from the institutional ethical clearance committee of the Aklilu Lemmaa Institute of Pathobiology (ALIPB), Addis Ababa University. In addition, permission to proceed with the study was obtained from concerned bodies of the SRS including the regional health bureau and administrative bodies of the Shinile Wereda which includes different Kebeles. An oral consent for interview was obtained from each of the interviewee after explaining the purpose of the study and confidentiality and participatory features of the study to the study participants. Every study participant was interviewed independently and the collected information was kept confidential. During the training, the interviewers were told to respect the respondents’ right to decline to participate or to elect to discontinue the interview.

## Results

### Background characteristics

The socio-demographic profile of the respondents is summarized in Table 1. The mean age of the study participants was 33.4 years and their age ranged between 18–87 years. Male respondents constituted 50.2%, 54.6% were younger than 30 years of age, 79.0% belongs to the Somali ethnic group, 89.0% were Muslims, 48.5% were currently unmarried, 38.8% were students and 52.9% live on an irregular income.

**Table 1:** **Socio-demographic characteristic of the respondents in Shinile town, Somali regional state, eastern Ethiopia from January to May, 2013**

Characteristic	Number	Percentage
**Gender**		
Male	206	50.2
Female	204	49.8
**Age (years)**		
18-29	224	54.6
30-44	99	24.2
45-59	42	10.2
60+	45	11.0
**Marital status**		
Married	184	44.9
Unmarried	199	48.5
Other	27	6.6
**Ethnicity**		
Somali	324	79.0
Other	86	21.0
**Religion**		
Muslim	365	89.0
Other	45	11.0
**Occupational status**		
Employed	139	33.9
Unemployed	112	27.3
Student	159	38.8
**Educational status**		
Illiterate	116	28.3
Less than grade 8	46	11.2
Grade 8-grade 12	129	31.5
Higher education	119	29.0
**Family income**		
No defined income	135	32.9
Irregular income	217	52.9
Regular income	58	14.2

### Knowledge about the cause, symptoms, mode of transmission, prevention and treatment of TB

**Figure 1:**
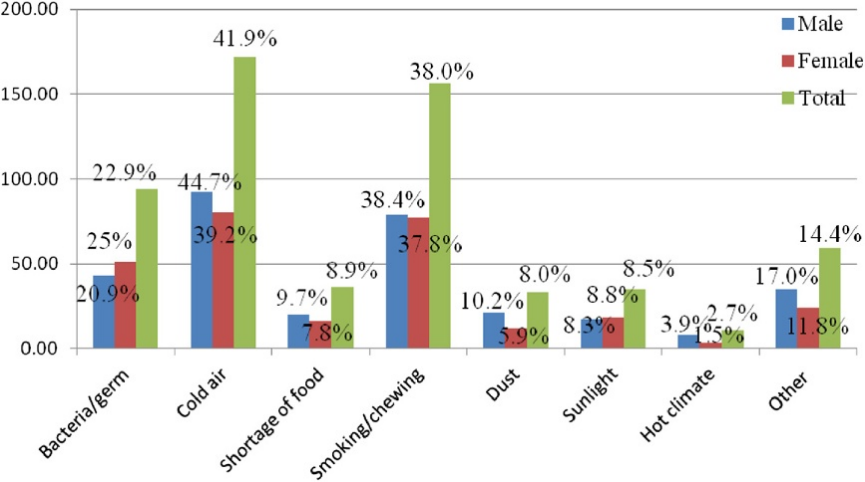
**Communities’ knowledge about the cause of TB in Shinile town, Somali regional state, eastern Ethiopia from January to May, 2013.**

An overwhelming majority (94.9%) had, at the very least, heard of TB disease without significant gender difference. The main sources of information about TB in the study area include mass media (particularly radio) and health professionals (Table 2). Among the study participants, 22.9% answered *“M. tuberculosis”* or *“bacteria/germs”* for the question *“what is the cause of TB?”* Other responses were cold air (42%), smoking or chat chewing (38.1%), shortage of food (8.9%), sun light (9.0%), and dust (8.0%) (Figure 1).

**Table 2:** **Communities’ source of information about TB in Shinile town, Somali regional state, eastern Ethiopia from January to May, 2013**

Variables	Number (%) of male	Number (%) of female	Total number (%)
**Heard of TB**			
Yes	199 (96.6)	190 (93.1)	389 (94.9)
No	7 (3.4)	14 (6.9)	21 (5.1)
**Sources of information**			
Newspaper and magazines	27 (13.1)	28 (13.4)	55 (13.4)
Media (radio)	135 (65.5)	124 (60.8)	259 (63.2)
Brochures, posters, etc.	16 (7.8)	14 (6.9)	30 (7.3)
Health workers	123 (59.7)	110 (53.9)	233 (56.8)
Family, friends,	59 (28.6)	48 (23.5)	107 (26.1)
Religious leader	24 (11.6)	19 (9.3)	43 (10.5)
Teachers	76 (36.9)	48 (23.5)	124 (30.2)

With respect to the communities’ knowledge about the signs and symptoms of TB (Table 3), persistence cough for 2 or more weeks (72.4%) was the most commonly mentioned symptom of TB. Other symptoms mentioned by the respondents include coughing up sputum with blood (52.2%), and chest pain (29.0%). Majority (80.0%) knew that TB can be transmitted from a patient to another person and 15.6% didn’t know whether it can be transmitted or not. The most frequently mentioned possible modes of transmission were through the air when a person with TB sneezes or coughs (59.3%) and sharing cups with the patient (35.6%). Some people also mentioned sharing feeding utensils, living in the same room and other as a route of transmission of TB. Male participants were more likely to know TB as an air borne disease compared to females (64.6% vs 53.9%, p = 0.028).

**Table 3:** **Communities’ knowledge about the signs/symptoms, mode of transmission, prevention and treatment of TB in Shinile town, Somali regional state, eastern Ethiopia from January to May, 2013**

Knowledge variable	Number (%) of male	Number (%) of female	Total number (%)
**Signs/symptoms of TB**			
Cough for 2 weeks	158 (76.7)	139 (68.1)	297 (72.4)
Sputum with blood	117 (56.8)	97 (47.6)	214 (52.2)
Weight loss	56 (27.2)	53 (26.0)	109 (26.6)
Loss of appetite	37 (18.0)	34 (16.7)	71 (17.3)
Fever and sweat	32 (15.5)	26 (12.8)	58 (14.2)
Chest pain	60 (29.1)	59 (28.9)	119 (29.0)
Other (skin rash, etc.)	3 (1.5)	0 (0.0)	3 (0.7)
Don’t know	4 (1.9)	2 (1.0)	6 (1.5)
**Can TB be transmitted?**			
Yes	169 (82.0)	157 (80.0)	326 (80.0)
No	8 (3.9)	12 (5.9)	20 (4.9)
Don’t know	29 (14.1)	35 (17.2)	64 (15.6)
**TB transmitted through**			
Hand shaking	8 (3.9)	12 (5.9)	20 (4.9)
Cough/breath	133 (64.6)	110 (53.9)	243 (59.3)
Sharing cups	74 (35.9)	72 (35.3)	146 (35.6)
Sharing feeding material	34 (16.5)	35 (17.2)	69 (16.8)
Touching items in public	19 (9.2)	11 (5.4)	30 (7.3)
Other (crowding, poor hygiene )	2 (1.0)	3 (1.5)	5 (1.2)
Don’t know	3 (1.5)	4 (2.0)	7 (1.7)
**Can TB be prevented?**			
Yes	165 (80.1)	160 (78.4)	325 (79.3)
No	10 (4.9)	8 (3.9)	18 (4.4)
Don’t know	31 (15.1)	36 (17.7)	67 (16.3)
**Preventive methods of TB**			
Avoid shaking hand	5 (2.4)	6 (2.9)	11 (2.7)
Covering mouth	97 (47.1)	89 (43.6)	186 (45.4)
Avoid sharing cups	68 (33.0)	66 (32.4)	134 (32.7)
Early treatment	57 (27.7)	60 (29.4)	117 (28.5)
Good nutrition	28 (13.6)	22 (10.8)	50 (12.2)
Using separate rooms	94 (45.6)	71 (34.8)	165 (40.2)
Closing window	19 (9.2)	21 (10.3)	40 (9.8)
Other (good hygiene, etc.)	0 (0.0)	4 (2.0)	4 (1.0)
Don’t know	3 (1.5)	2 (1.0)	5 (1.2)
**Is TB treatable?**			
Yes	182 (88.4)	178 (87.3)	360 (87.8)
No	5 (2.4)	3 (1.5)	8 (2.0)
Don’t know	19 (9.2)	23 (11.3)	42 (10.2)
**Treatment method of TB**			
Herbal remedies	18 (8.7)	25 (12.3)	43 (10.5)
Home rest without treatment	12 (5.8)	13 (6.4)	25 (6.1)
Pray	30 (14.6)	36 (17.7)	66 (16.1)
Modern drug	143 (69.4)	136 (66.7)	279 (68.1)
Self-treatment	75 (36.4)	76 (37.3)	151 (36.8)
Other (respecting elders, etc.)	1 (0.2)	2 (0.5)	3 (0.8)
Don’t know	0 (0.0)	2 (1.0)	2 (0.5)

Most (79.3%) of the participants responded that transmission of TB would be preventable (Table 3). When inquired about the preventive measures for TB, responses included covering mouth and nose when coughing or sneezing, use separate room for the patient, avoid sharing cups with the patient, and early treatment. Relatively, a higher proportion of males were in favor of using separate room for TB patients as a prevention method of the disease than female individuals (45.6% versus 34.8%, p = 0.025). vs 34.8%, p = 0.025). Majority (87.8%) of the respondents knew that TB is treatable disease (Table 3) and responses regarding treatment included modern drugs given by health personnel in health institutions (68.1%), self-treatment (36.8%), praying, herbal remedies and home rest.

Overall knowledge mean score about TB was 10.67. One hundred and eighty seven participants (45.6%) had low overall TB knowledge and 223 (54.4%) had high overall knowledge about TB. Overall knowledge about TB was not significantly associated with gender. But, high level of overall knowledge about TB was reported among individuals with educational status of grade 8 up to grade 12 compared to illiterate individuals (adjusted OR, 2.33; 95% CI, 1.18 to 4.62; p value < 0.001) and among individuals with regular income compared to individuals who had no definite income (adjusted OR, 3.89; 95% CI, 1.67 to 9.08; p value <0.05).

Mean knowledge scores of the study participants about the signs/symptoms, modes of transmission and prevention of TB were 2.12, 3.53 and 3.23, respectively. Majority (73.9%) had scored good knowledge and 26.1% scored low level of knowledge about the signs and symptoms of TB. One hundred and fifty nine (38.8%) had low level of knowledge and 251 (61.2%) had high level of knowledge about the transmission method of the disease. With regards to the modes of prevention of TB, 42.2% of the study participants had low level of knowledge and 57.8% had scored high level of knowledge. High knowledge score about the effective treatment of TB was also reported by 68.1% as being modern drugs given by health personnel in health institutions.

### Communities’ attitudes and practices towards TB

Communities’ attitude towards TB disease in the current study area is summarized in Table 4. More than half of the study participants considered TB as a very serious disease and 40.2% said that TB is a very serious problem in their area. For the question “*do you think you can get TB*?” 52.9% answered “*yes*”, 33.4% said “*don’t know*” and the response of 13.7% was “*no*”. With regards to their reaction if they themselves had TB, most of them responded that they would experience fear, while others replied that they would experience sadness and/or hopelessness. Male participants were more likely to feel ashamed of being a TB patient compared to female participants (12.6% versus 4.9%, p = 0.006).

**Table 4:** **Communities’ attitude about TB in Shinile town, Somali regional state, eastern Ethiopia from January to May, 2013**

Variables	Number (%) of male	Number (%) of female	Total number (%)
**How serious a disease is TB?**			
Very serious	117 (56.8)	110 (53.9)	227 (55.4)
Somewhat serious	27 (13.1)	23 (11.3)	50 (12.2)
Not very serious	16 (7.8)	10 (4.9)	26 (6.3)
Don’t know	46 (22.3)	61 (29.9)	107 (26.1)
**How serious a problem do you think TB is in your area?**			
Very serious	88 (42.7)	77 (37.7)	165 (40.2)
Somewhat serious	37 (18.0)	32 (15.7)	69 (16.8)
Not very serious	27 (13.1)	27 (13.2)	54 (13.2)
Don’t know	54 (26.2)	68 (33.3)	122 (29.8)
**Do you think you can get TB?**			
Yes	110 (53.4)	107 (52.5)	217 (52.9)
No	33 (16.0)	23 (11.3)	56 (13.7)
Don’t know	63 (30.6)	74 (36.3)	137 (33.4)
**What would be your reaction if you were found out that you have TB?**			
Fear	139 (67.5)	145 (71.1)	284 (69.3)
Surprised	14 (6.8)	13 (6.4)	27 (6.6)
Shame	26 (12.6)	10 (4.9)	36 (8.8)
Sadness or hopelessness	41 (19.9)	35 (17.2)	76 (18.5)
Other (scares, don’t know,don’t fear, etc.)	30 (14.7)	30 (14.6)	60 (14.6)
**Some people more likely to become infected with TB than others?**			
Yes	137 (66.5)	133 (65.20)	270 (65.9)
No	26 (12.6)	30 (14.70)	56 (13.7)
Don’t know	43 (20.9)	41 (20.10)	84 (20.5)
**If yes, who is more likely to be infected?**			
Men	45 (32.9)	37 (27.8)	82 (30.4)
Women	48 (35.0)	44 (33.1)	92 (34.1)
Both men and women	18 (13.1)	20 (15.0)	38 (14.1)
Children <5 years	45 (32.9)	49 (36.8)	94 (34.8)
Very old people	112 (81.8)	102 (76.7)	214 (79.3)

About 42.4% of the participants had no particular feeling towards people with TB disease and 39.0% felt compassion and desire to help the patient (Table 5). They were also asked on how a person who has TB usually regarded/treated in their community and 51.7% of them responded that the community mostly supports and helps the patient, about 31.5% replied that they don’t know the communities’ feeling towards a TB patient or not sure whether they help or not. Only sixteen respondents (3.9%) replied that most people reject the patient. Among those, 65.9% who suggested that some people are more likely to become infected with TB than others, a considerable number (79.3%) perceived that very old people are more prone to TB.

**Table 5:** **Assessment of communities’ stigma towards TB & TB patients in Shinile town, Somali regional state, eastern Ethiopia from January to May, 2013**

Variables	Number (%) of male	Number (%) of female	Total number (%)
**Do you know people who have/had TB?**			
Yes	123 (59.7)	124 (60.8)	247 (60.2)
No	41 (19.9)	41 (20.1)	82 (20.0)
Don’t know	42 (20.4)	39 (19.1)	81 (19.8)
**How is your feeling towards people with TB disease?**			
“I feel compassion and desire to help.”	88 (42.7)	72 (39.3)	160 (39.0)
“I feel compassion but stay away from them.”	9 (4.4)	15 (7.4)	24 (5.9)
“It is their problem and I cannot get TB.”	1 (0.5)	3 (1.5)	4 (1.0)
“I fear them because they may infect me.”	12 (5.8)	15 (7.4)	27 (6.6)
“I have no particular feeling.”	89 (43.2)	85 (41.7)	174 (42.4)
**In your community, how is a person who has TB usually regarded/treated?**			
Most people reject him or her	9 (4.4)	7 (3.4)	16 (3.9)
Most people are friendly, but they generally try to avoid him or her	11 (5.3)	20 (9.8)	31 (7.6)
Mostly supports and helps him or her	103 (50.0)	109 (53.4)	212 (51.7)
Other (don’t know their feeling, not sure whether they help or not, don’t give special attention, etc.)	75 (36.4)	54 (26.5)	129 (31.5)

TB related health care seeking behaviors and practices of communities in the current study area are summarized in Table 6. Significant proportion of the study participants (66.3%) said that they would consult doctors or other medical workers about their illness if they got TB, while others would like to talk to their parents, close friends, religious leaders and elders or community leaders. When asked what they would do if they had symptoms of TB, 71.0% answered that they would go to a health facility. Other mentioned pursuing other self-treatment options (like herbs), visiting traditional healers, praying and visiting a pharmacy. Medical help would be sought immediately by 39.3% of the study respondents as soon as they realized that they had symptoms related to TB. Others responded in such a way that they would seek medical help when self-treatment failed to work or if symptoms that look like TB signs lasted for 3–4 weeks, while 47 (11.5%) didn’t know what to do.

**Table 6:** **Communities’ practice about TB in Shinile town, Somali regional state, eastern Ethiopia from January to May, 2013**

Variables	Number (%) of male	Number (%) of female	Total number (%)
**Who would you talk to about your illness if you had TB?**			
Doctor or other medical worker	145 (70.4)	127 (62.3)	272 (66.3)
Spouse	8 (3.9)	6 (2.9)	14 (3.4)
Parent	87 (42.2)	86 (42.2)	173 (42.2)
Close friend	44 (21.4)	40 (19.6)	84 (20.5)
No one	3 (1.5)	2 (1.0)	5 (1.2)
Other (religious leaders, elders, etc.)	34 (16.5)	30 (14.7)	64 (15.6)
**What would you do if you thought you had symptoms of TB?**			
Go to health facility	151 (73.3)	140 (68.6)	291 (71.0)
Go to pharmacy	11 (5.3)	18 (8.8)	29 (7.1)
Go to traditional healers	39 (18.9)	33 (16.2)	72 (17.6)
Pursue self-treatment options (herbs, etc.).	66 (32.0)	66 (32.4)	132 (32.2)
Other (go to mosque, pray, etc.)	22 (10.7)	28 (13.7)	50 (12.2)
**If you had symptoms of TB, at what point would you seek medical help?**			
When treatment on my own does not work	69 (33.5)	67 (32.8)	136 (33.1)
When TB symptoms last for 2 or more weeks	26 (12.6)	22 (10.8)	48 (11.7)
As soon as I realize TB symptoms	82 (39.8)	79 (38.7)	161 (39.3)
I would go to health facility or contact a doctor	10 (4.9)	8 (3.9)	18 (4.4)
I don’t know	19 (9.2)	28 (13.7)	47 (11.5)
**If you would not go to the health facility, what is the reason?**			
Not sure where to go	9 (4.5)	6 (3.2)	15 (3.9)
Cost	12 (6.0)	12 (6.3)	24 (6.2)
Difficulties with transportation/distance	123 (61.8)	109 (57.4)	232 (59.6)
Do not trust medical workers	21 (10.6)	17 (9.0)	38 (9.8)
Do not like attitude of medical workers	11 (5.53)	9 (4.7)	20 (5.1)
Cannot leave my work (overlapping work hours with medical facility working hours)	17 (8.5)	11 (5.8)	28 (7.2)
Do not want to find that something is wrong	54 (27.1)	43 (22.6)	97 (24.9)
Other (no reason, etc.)	20 (10.1)	25 (13.2)	45 (11.6)

Mean knowledge score about the health care seeking behaviors and practices about TB was 2.4. One hundred and fifty nine (38.78%) had poor knowledge and 251(61.2%) had high knowledge about health care seeking behaviors and practices about the disease. Mean knowledge score about attitude towards TB was 3.76. One hundred and seventy six (42.9%) had poor attitude and two hundred and thirty one (57.1%) had good attitude towards TB and those affected by the disease.

Table 7 shows the results of the multivariate analysis while assessing the effect of socio-demographic characteristics on the overall knowledge of study participants about TB. Higher level of overall TB knowledge about TB was associated with having regular income compared with not having definite income (adjusted OR, 3.9; 95% CI, 1.7 to 9.1; p =0.007) and with educational status being between grade 8 up to grade 12 compared to being illiterate (adjusted OR, 2.3; 95% CI, 1.2 to 4.6; p < 0.001) High level of overall knowledge about TB was not significantly associated with the gender of the study participants.

**Table 7:** **Association of respondents’ socio-demographic characteristics with respondents’ overall knowledge of TB in Shinile town, Somali regional state, eastern Ethiopia from January to May, 2013**

Characteristic	Number (%) having high overall TB knowledge	Crude OR (95%, CI)	Adjusted OR (95%, CI)
**Gender**			
Male	118 (57.3)	Reference	Reference
Female	105 (51.5)	0.79 (0.53-1.2)	0.9 (0.6-1.4)
**Age (years)**			
18-29	146 (65.2)	Reference	Reference
30-44	38 (38.4)	0.3 (0.2-0.5)	0.5 (0.3-0.8)
45-59	15 (35.7)	0.3 (0.2-0.6)	0.5 (0.2-1.1)
60+	24 (53.3)	0.6 (0.3-1.2)	1.2 (0.5-2.6)
**Occupation**			
Employed	63 (46.7)	Reference	Reference
Unemployed	48 (40.5)	1.0 (0.6-1.6)	1.0 (0.8-1.7)
Student	109 (66.9)	2.3 (1.4-3.7)	1.9 (0.9-3.9)
**Educational status**			
Illiterate	43 (37.1)	Reference	Reference
< than grade8	20 (43.3)	1.3 (0.7-2.6)	1.2 (0.6-2.6)
Grade8-grade12	84 (65.1)	3.2 (1.9-5.3)	2.3 (1.2-4.6)
Higher education	76 (63.9)	3.0 (1.8-5.1)	2.0 (1.0-4.0)
**Family income**			
No defined income	65 (48.2)	Reference	Reference
Irregular income	116 (53.5)	1.2 (0.8 - 1.9)	2.2 (1.3-3.7)
Regular income	42 (72.4)	2.8 (1.5 - 5.5)	3.9 (1.7-9.1)

## Discussion

This study showed that TB is familiar to the general community in the current study area, as the majority (94.9%) of the study participants had indicated that they have heard of TB disease, which is similar to previous studies done among pastoral communities in the Shinile area [16] and middle and lower Awash valley of Afar region, Ethiopia [29], where 92.8% and 95.6% of the study participants were aware of the disease, respectively. However, in accordance with earlier studies in Somali region [16, 34] and southwest Ethiopia [31] as well as in Afar region [29], Kenya [35], and Pakistan [36], the respondents had limited information concerning bacteria as a causative agent of TB. Instead, most of them perceived mainly either cold air or smoking and chat chewing as the cause of TB, which is more or less similar with other studies [29, 30, 37]. Poor awareness regarding etiology of the disease may has a negative impact on patients’ attitude towards health-seeking behavior and preventive methods as most people with such believes may not visit health facilities or they may consider various traditional alternatives.

Based on the results of this study, the respondents had basic knowledge about the common signs/symptoms of TB and its modes of transmission, which agrees with previous studies in a rural community in southwest Ethiopia [31], in northeast Ethiopia [29], and also in Iran [38] and Philippines [39]. In this regard, it was reported that persistence cough for 2 or more weeks, coughing up sputum with blood, chest pain and weight loss were the common sign and symptom of TB. Through the air when a person with TB sneezes or coughs, and sharing cups with the patient were the common perceived modes of transmission in different studies [29, 40, 41]. The reported basic communities’ knowledge about the symptoms and transmission methods of TB has an important implication for the TB control program in the current study area in particular and also in the country in general in that it could reduce diagnosis and treatment delay, as well as the spread of the disease.

Another important aspect noted in this study was that most of the participants were aware of the prevention and treatment methods of TB, which is more or less similar to a study performed by Melaku et al. [16]. Accordingly, covering mouth and nose when a person with TB coughs or sneezes, using a separate room for the patient, avoid sharing cups with the patient, early treatment and good nutrition as a prevention methods were similarly documented by earlier studies from Ethiopia [16, 31] and also from Pakistan [40]. Furthermore, respondents’ knowledge regarding treatment of the disease using modern drugs was very high compared to the results of previous studies conducted in other parts of Ethiopia [30, 31]. It is interesting to note, however, that association of self-treatment options, traditional healers and praying to the treatment mode of the disease cannot be neglected, which is in consistence with findings by Deribew et al. [30]. This may be due to, they may not suspect TB upon appearance of early symptoms (cough, fever, etc.) unless severe symptoms (hemoptysis, weight loss, etc.) set in, which can be evidenced by finding of Gele et al. [34] as the Somali pastoralists consider persistent cough a normal phenomenon, not as a potential symptom of TB. The other reason could lie in the strong belief in spiritual healers in Somali society.

In this study, high overall TB knowledge was significantly associated with monthly income status and educational status of the study participants. The findings corroborate previous studies in Shinile [16], southwest Ethiopia [30] and elsewhere [42]. Therefore, public awareness programs using media are crucial in educating the masses, and moreover, health education program to raise communities’ knowledge of TB is mandatory.

More brave was the fact that majority (71.0%) of the study participants in this survey reported that they would go to health facility if they thought they had symptoms of TB in contrast to earlier studies in Somali region, Ethiopia [14, 34] and also in Kenya [35]. Moreover, most respondents would seek medical help immediately as soon as they realize they had symptoms related to TB, unlike other reports from the country [15, 34]. Increased involvement of Health Extension Workers in expanding health education delivery may have an impact in raising communities’ awareness towards TB in the area.

It was also found out that participants from the current study area considered that TB is a very serious disease in general and a very serious problem in their area. Furthermore, majority of the respondents indicated that they would feel fear or scare and sadness or hopelessness if they found they have TB. Similar feelings have been associated with TB in Pakistan [36, 40]. On the other hand, a high proportion of the study subjects had no particular feeling towards people with TB disease, which means that there is no discrimination against TB patients in the current study area. Moreover, more than half the study subjects said that TB patients are mostly supported and helped by the community. This is in contrast to many other studies conducted in the country [16, 30, 37] and as well as in Kenya [35], India [41] and Pakistan [36, 40]. The perception of TB as a very dangerous disease resulting in fear might be due to the factors: relatively long time needed for its treatment, its mortality in the community, the coughing up of blood associated with many afflicted by the disease and comparison of TB with incurable tumors and cancer.

### Limitations

In this study, efforts were made to assess the knowledge, attitudes and practices of community towards TB which could support the TB control programs in the Somali region in particular and Ethiopia in general. However, the study has potential limitations including lack of focus group discussion which might be used to triangulate the findings, absence of information on HIV, lack of questions about MDR and XDR-TB, and lack of data about current and former personal TB infection or family members with current and former TB infection.

## Conclusion

Generally, the community in Shinile town had basic awareness about TB. However, they had little information about the cause of TB, as a significant number of the participants perceived that cold air or smoking as the cause of the disease. It should be also noticed that a considerable number of participants were in favor of using self-treatment options as an effective treatment method of the disease. Therefore, a strategy (health education) directed towards bringing a significant change in their knowledge specifically about the causative agent of TB, and means of transmission, prevention, and treatment is essential.

## Electronic supplementary material

Additional file 1: **Questionnaires administered in the study.doc, 25 K.** The questionnaire has all the questions that were used to collect data reported within the manuscript. (DOCX 25 KB)

## Abbreviations

AOR: Adjusted odds ratio
CI: Confidence interval
MDR-TB: Multi drug resistance TB
OR: Odds ratio
SRS: Somali Regional State
SRSHB: Somali Regional State Health Bureau
TB: Tuberculosis
WHO: World health organization
XDR-TB: Extensive drug resistance TB.
