# Supplementary material for: Community knowledge, attitude, and practices towards tuberculosis in Shinile town, Somali regional state, eastern Ethiopia: a cross-sectional study
Source: BMC Public Health. 2014 Aug 7;14:804. doi: 10.1186/1471-2458-14-804 (PMC4133079; doi:10.1186/1471-2458-14-804)
Supplement: Supplementary file 1 — Additional file 1: Questionnaires administered in the study.doc, 25 K. The questionnaire has all the questions that were used to collect data reported within the manuscript. (DOCX 25 KB) [file 12889_2014_6923_MOESM1_ESM.docx]

**Questionnaire for assessment of Knowledge, Attitude, and Practice of communities towards tuberculosis in Shinille Town, Somali Regional State, Eastern Ethiopia from February to May, 2013.**

Date: ___ / ___ / ___

**PART ONE: General and socio-demographic characteristics of the respondents**

1. **code of the respondent ___________________**
2. **Gender: A.** Male B. Female
3. **Age (year):** ____________________________
4. **To which ithic group do you belong?**
5. Somali B. Amhara C. Oromo D. Tigre E. Other__________
6. **What is your religion?**
7. Orthodox B. Muslim C. Protestant D. Catholic E. Other _________
8. **What is your current marital status?**
9. Married B. Unmarried. C. Divorced D. Widowed
10. **What is your current educational status?**
11. Illitrate D. University level
12. Less than grade E. Other ____________________
13. Grade 8-grade
14. **What is your current occupation?**
15. Employed C. Housewife E. Unskilled worker G. Student
16. Un-employed D. Farmer F. Daily wages H. Other ____
17. **What is your average household income per month?**
18. No defined income B. Irregular income C. Regular income of

**PART TWO: Questionnaire about TB knowledge and awareness**

1. **Have you ever heard about the disease called TB?** A. Yes B. No
2. **If yes, from whom/where?**
3. Newspapers and magazines E. Family, friends, neighbours and colleagues
4. Media (radio, TV, etc.) F. Religious leaders
5. Brochures, Posters and Printed material G. Teachers
6. Health workers H. Other (please explain):____
7. **What do you think is the cause of TB**
8. Bacteria/Germs C. Shortage of food E. Hot climate G. Smoking/chewing
9. Cold air D. Dust F. Sunlight H. Other (specify)__
10. **What are some of the common signs and symptoms of TB?**
11. Cough for 3 or more weeks E. Fever and sweat at night
12. Sputum with blood F. Chest pain
13. Weight loss G. Don’t know
14. Loss of appetite H. Other (specify):_____
15. **Do you think that the disease can be transmitted from the patient to other person?**
16. Yes B. No C. Don’t know
17. **If yes, how can a person get TB?**
18. Through the air when a person with TB sneezes or coughs
19. Through touching items in public places (doorknobs, handles in transportation, etc,)
20. Through sharing cups E. Through handshakes F. Don’t know
21. Through eating from the same plate G. Other (specify)___
22. **Do you think that the transmission of TB is preventable?**
23. Yes B. No C. Don’t know
24. **If yes, how can a person prevent getting TB?**
25. Covering mouth and nose when coughing or sneezing
26. Avoid shaking hands F. Through good nutrition
27. Early treatment G. Use separate room for the patient
28. Avoid sharing cups with a patient H. Don’t know
29. Closing windows at home I. Other specify ______________
30. **Can TB be cured?**
31. Yes B. No C. Don’t know
32. **How can someone with TB be cured?**
33. Modern drugs given by health institutions health personals
34. Herbal remedies D. Praying F. Selftreatment
35. Home rest without medicine E. Don’t know G. Other ________

**PART THREE: Assessment of TB attitudes and health care-seeking behavior of Communities.**

1. **In your opinion, how serious a disease is TB?**
2. Very serious D. Not very serious
3. Somewhat serious E. Don’t know
4. **How serious a problem do you think TB is in your area?**
5. Very serious D. Not very serious
6. Somewhat serious E. Don’t know
7. **Do you think you can get TB?**
8. Yes B. No C. Don’t know
9. **What would be your reaction if you were found out that you have TB?**
10. Fear C. Shame E. Other ______
11. Surprise D. Sadness or hopelessnes
12. **Who would you talk to about your illness if you had TB?**
13. Doctor or other medical worker C. Parent E. No one
14. Spouse D. Close friend F. Other ____
15. **What would you do if you thought you had symptoms of TB?**
16. Persue other self-treatment options (herbs, etc.)
17. Go to health facility D. Go to traditional healers
18. Go to pharmacy F. Other ____________
19. **If you had symptoms of TB, at what point would you seek medical help?**
20. When treatment on my own does not work
21. When symptoms that look like TB signs last for 3-4 weeks
22. As soon as I realize that my symptoms might be related to TB
23. I would go to health facility or contact health personals.
24. I don’t know
25. **If you would not go to the health facility, what is the reason?**
26. Not sure where to go
27. Cost
28. Cannot leave work (overlapping work hours with medical facility working hours)
29. Do not want to find out that something is really wrong
30. Difficulties with transportation/distance to clinic
31. Do not trust medical workers
32. Do not like attitude of medical workers
33. Other (please explain):________________________________

**PART FOUR: Assessment of TB stigma of Community.**

1. **Do you know people who have/had TB?**
2. Yes B. No C. Don’t know
3. **How do you feel towards people with TB disease?**
4. “I feel compassion and desire to help.”
5. “I feel compassion but I tend to stay away from these people.”
6. “It is their problem and I cannot get TB.”
7. “I fear them because they may infect me.”
8. “I have no particular feeling.”
9. Other (please explain):______________________________
10. **In your community, how is a person who has TB usually regarded/treated?**
11. Most people reject him or her
12. Most people are friendly, but they generally try to avoid him or her
13. The community mostly supports and helps him or her
14. Other (please explain):____________________________________________
15. **In your opinion, are some people more likely to become infected with TB than others?**
16. Yes B. No C. Don’t know
17. **If yes, who is more likely to be infected?**
18. Men C. both men and women E. very old people G.otherspecify
19. Women D. children under 5 years F. don’t know

**Thank you very much for participating in my survey!!**
